# Machine Learning for the Diagnosis of Orthodontic Extractions: A Computational Analysis Using Ensemble Learning

**DOI:** 10.3390/bioengineering7020055

**Published:** 2020-06-12

**Authors:** Yasir Suhail, Madhur Upadhyay, Aditya Chhibber, Kshitiz

**Affiliations:** 1 Department of Biomedical Engineering, University of Connecticut Health Center, Farmington, CT 06032, USA; 2 Division of Orthodontics, School of Dental Medicine, University of Connecticut Health Center, Farmington, CT 06032, USA; maupadhyay@uchc.edu; 3 Private Practice, Norwalk, OH 44857, USA; adityachhibber14@gmail.com

**Keywords:** orthodontics, neural network, machine learning, random forests, ensemble methods

## Abstract

Extraction of teeth is an important treatment decision in orthodontic practice. An expert system that is able to arrive at suitable treatment decisions can be valuable to clinicians for verifying treatment plans, minimizing human error, training orthodontists, and improving reliability. In this work, we train a number of machine learning models for this prediction task using data for 287 patients, evaluated independently by five different orthodontists. We demonstrate why ensemble methods are particularly suited for this task. We evaluate the performance of the machine learning models and interpret the training behavior. We show that the results for our model are close to the level of agreement between different orthodontists.

## 1. Introduction

Extraction of teeth is one of the most critical and controversial decisions in orthodontic treatment, largely because extractions are irreversible [1,2]. These decisions are based on clinical evaluations, patient photographs, dental study models, radiographs, and substantially rely upon the experience and knowledge of the clinician. A wrong decision can lead to undesirable results like suboptimal esthetics, improper bite, functional abnormalities related to mastication and speech, and in the worst-case scenario, an unfinished treatment. To date, the decision to extract teeth is not formalized and standardized, and depends upon the practitioner’s heuristics [3]. This often causes intra-clinician and inter-clinician variability in the decision-making process [4,5]. Therefore, for hundreds of students, residents, orthodontists, and dentists across the globe, diagnosis and treatment planning poses a significant challenge. The resultant gap in the knowledge or data interpretation can be critical. Therefore, in order to standardize the decision-making process, newer approaches are required.

In this study, we aim to create an artificial intelligence decision-making model for the diagnosis of extractions using neural network machine learning. The primary objectives of the study were (1) to develop a decision-making model that simulates experts’ decisions of whether a tooth needs to be extracted or not based on standardized orthodontic pretreatment records (patient photographs and X-rays), and (2) to determine the knowledge elements required in formulating orthodontic extraction/non-extraction treatment decisions. It was expected that the diagnostic model created would match an expert’s diagnosis, both in binary decision-making (extraction vs. non-extraction outcomes), and in the more resolved decision-making process of which a specific extraction outcome would be followed (out of the 13 possible outcomes). This method would not only limit variability in decision-making in orthodontics but also limit the adverse effects of wrongly prescribed tooth extraction protocols. Additionally, this could also serve as a testing tool to train dentists and orthodontic students. 

Orthodontic pretreatment records in the form of extraoral photos, intra-oral photos, and cephalometric X-rays were collected. A panel of experienced orthodontists (also henceforth referred to as experts) evaluated the records individually and predicted the final outcome of extraction/non-extraction.

## 2. Materials and Methods

### 2.1. Data Collection and Feature Selection

The data consisted of 300 pretreatment patient records obtained from a private practice in Norwalk, Ohio, USA (orthodontist: C.A). Medical charts and conventional diagnostic records such as lateral head films (cephalometric X-rays), panoramic radiographs, facial photographs, and intraoral photographs were employed for each subject and screened by C.A for completeness. All subjects had full permanent dentitions except for the third molar, no abnormalities of the craniofacial forms or skeletal deformities, and no history of orthodontic treatment. Nineteen feature variables or elements that characterize orthodontic problems and are assumed to be important in deciding whether or not teeth need to be extracted were selected. This selection was based on the existing orthodontic literature. For all subjects, 5 experts (C.A, V.M, D.S, C.P.J,), with an average experience of approximately 9 years among them, examined the records of each patient based on the pre-selected feature variables. Each expert also recorded his/her two most likely diagnostic outcomes (out of 14 available options) and categorized them as primary treatment and alternate treatment.

The data were compiled and evaluated for potential errors by one of the authors (U.M). Data sets for thirteen patients were eliminated due to incomplete records and errors in data recording.

### 2.2. Computational Analysis 

**Figure 1:**
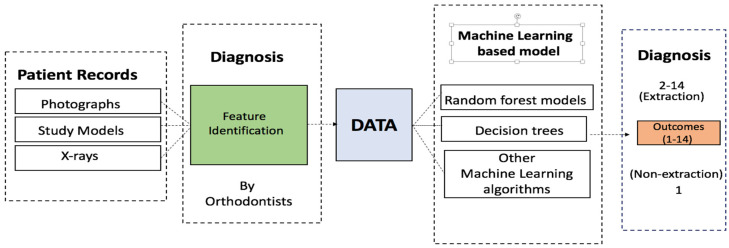
Schematic of the procedure followed in this work, from data collection to the machine learning diagnosis.

Expert-provided features and decision data were analyzed using the R [6] platform. The neural network model was built using the nnet [7] package, while the random forests were built and evaluated using the RandomForest [8] package. All calculations were performed using 5-fold cross-validation. The same cross-validation sets were used for each model and hyperparameter determination. Figure 1 shows the schematic for the data collection and computational analysis.

## 3. Results

**Figure 2:**
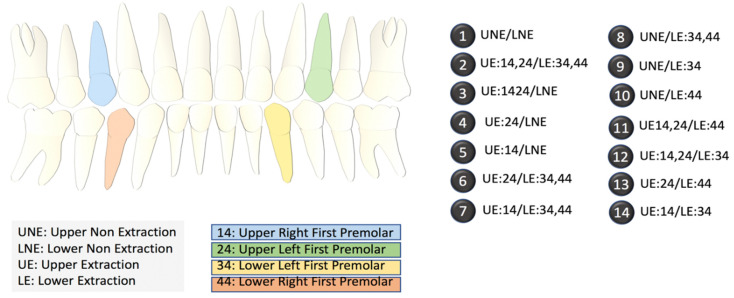
Index of the different extraction options. The diagram on the left shows the locations of the upper and lower premolars. The 14 options on the right list the specific extraction procedures in terms of the locations of the teeth. NE refers to no extraction.

We collected data for 287 patients from five different experts. Each expert assigned values to 19 pre-selected diagnostic features based on cephalometric images and patient photographs in addition to selecting a primary and alternate treatment option. Experts were allowed to decide between one of the two binary outcomes: non-extraction, or extraction. Within the extraction plan, depending upon which tooth/teeth required extraction, the experts had to select one (specific) outcome out of the 13 different options (2–14) provided (Figure 2). Crucially, the experts also opined on the second most preferred outcome (termed alternative outcome), which, considering the variability between experts’ opinions, allowed us to test the accuracy of our outcomes in a more robust manner.

### 3.1. Exploratory Analysis

**Figure 3:**
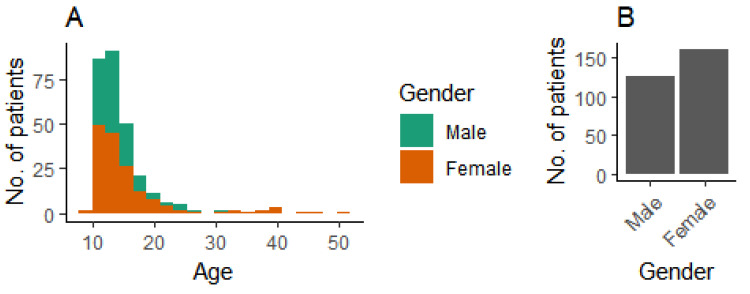
Demographic background of patients. (**A**) Age distribution, and (**B**) gender distribution.

We used patient data from 287 patients. The age and gender distributions are shown in Figure 3. 

First, we wanted to establish the degree of agreement between the experts who evaluated the patients included in this study. If the multiple treatment plans selected by the different experts are considered as the gold standard for a machine learning method, the inter-expert agreement should provide us a practical higher limit on the accuracy to achieve. The agreement on the primary outcome of treatment between the different experts varied from 65% to 71% (Table 1), and agreement on either the primary or alternative outcome varied from 93% to 98% (Table 2). These data highlight that different experts, well-trained in orthodontics, could defer in their primary opinions in some aspects.

### 3.2. Machine Learning Models

#### 3.2.1. Single Classifiers

**Figure 4:**
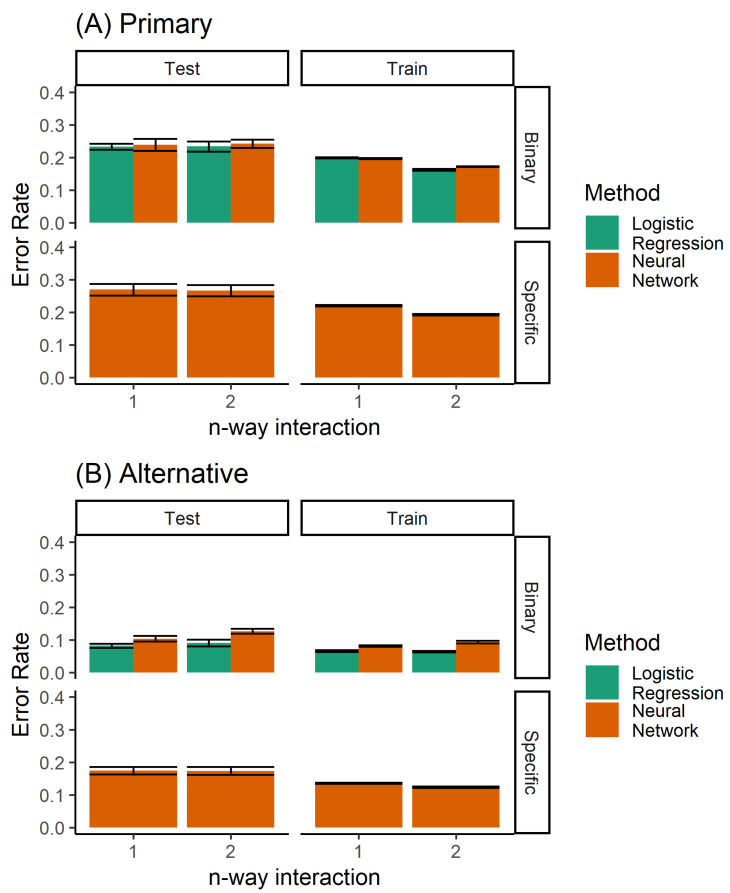
Performance of the single classifiers, when considering (**A**) only the primary diagnosis, and (**B**) both the primary and alternative.

A number of different methods can be used to build a classifier for the prediction of orthodontic extractions. We considered twin problems of predicting whether to extract teeth or not, and the specific extraction treatment plan. As a classification problem, we had a discrete prediction, and used a neural network to learn the multinomial regression. Each output neuron learns to predict a specific extraction, taking inputs from the raw data. No hidden units were used. In addition, logistic regression was used for predicting the binary decision of extraction/non-extraction. Figure 4 shows the performance of the logistic regression and the multinomial regression neural network model. The logistic regression model, by definition, was not able to predict the specific extraction procedure. However, for the binary problem (extraction/non-extraction), the logistic regression outperformed the multinomial trained neural network.

**Figure 5:**
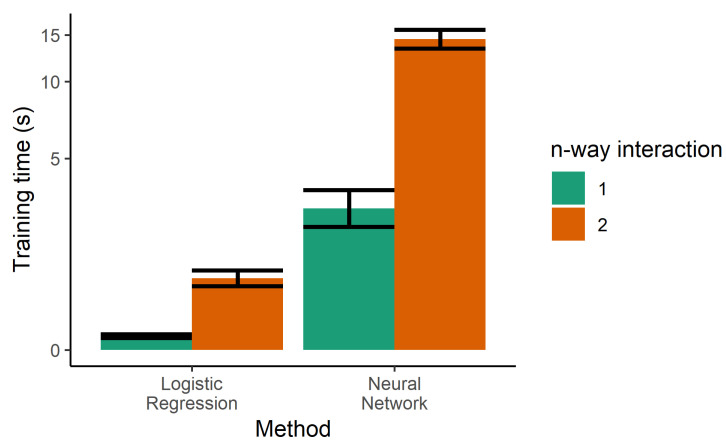
Training time for the single classifiers. Logistic regression was also trained with product terms (i.e., two-way interactions), dramatically increasing the training time. Due to the large dynamic range needed on the *y*-axis, it is drawn in the pseudolog transform.

The next step towards increasing the performance of the model was the use of two-way interactions in the logistic regression. Every pair of features was multiplied and used as additional features, generating a larger number of parameters. Although this was helpful in decreasing the error rates of the training sample, it increased the error of the test set, indicating that increasing the complexity of the model led to overfitting. Two-way interactions are more prone to overfitting due to the higher number of tunable parameters, and also increase the training time, as seen in Figure 5.

**Figure 6:**
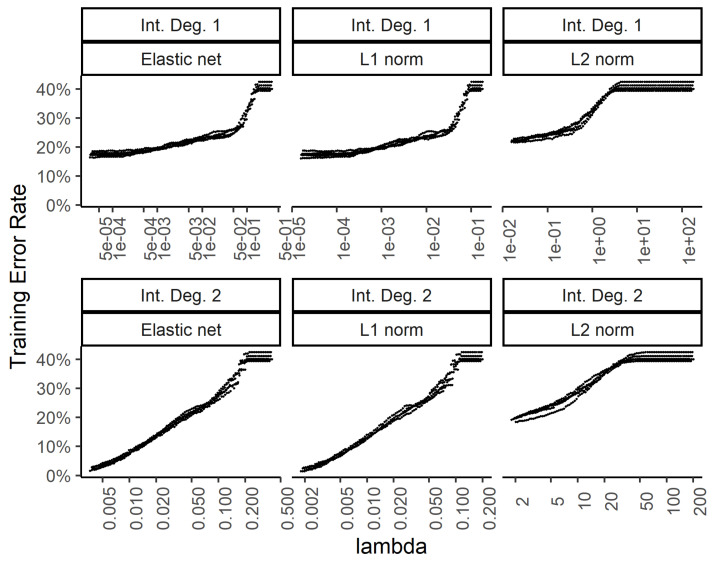
Effect of the weight regularization in the neural network/multinomial regression model on the training set error. The *x*-axis shows the regularization weight. The top row is for the neural network utilizing the raw input features while the bottom row is for using two-way interactions. The 3 columns show the results for the weight regularization penalty term formulated as an elastic net, L1 norm, and L2 norm.

**Figure 7:**
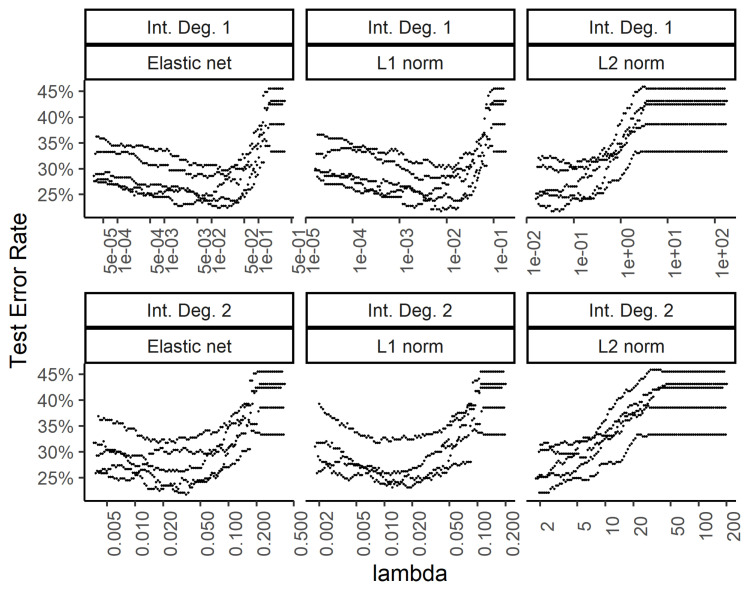
Effect of the weight regularization in the neural network/multinomial regression model on the test set error. The *x*-axis shows the regularization weight, with rows and columns corresponding to the interactions and weight regularization method as in Figure 6.

In order to explore the benefit of including higher order interactions against overfitting with too many parameters, we implemented weight regularization for the two-layer dense neural network. We added a weight regularization penalty for the L1 norm, L2 norm, and an equally weighted L1 and L2 norm as an elastic net. Figure 6 and Figure 7 show the training and test errors, respectively, for the three regularization schemes against the regularization weight.

While the training errors (Figure 6) monotonously increase with higher regularization weights for the L1, L2, and combined (elastic net) norms, the test error rates show a classic dip for the intermediate regularization weights (Figure 7). The addition of second-degree interactions in the predictors drives the training error to almost zero (bottom row of Figure 6), while the test error increases somewhat. This overfitting however, is countered by regularization.

#### 3.2.2. Random Forest as an Ensemble Classifier

Since the addition of additional parameters in the classifier (as seen in the logistic regression with multiplicative terms) leads to overfitting, we used an ensemble of classifiers to improve the performance. Ensemble methods are known to be resistant to overfitting. We trained random forest models using the standard algorithm, and varied the main hyperparameters to gain insight into the limitations for the performance.

Each decision tree in the random forest was constructed using a dataset sampled with replacement from the training set. This process of bagging is one of the ways in which each decision tree attempts to capture a different aspect of the data. During the construction of each decision tree, a small number of features were randomly selected at each level and the one that was the most discriminating among the classes was used. The process continued until each node contained no more than a specific minimal number of samples.

**Figure 8:**
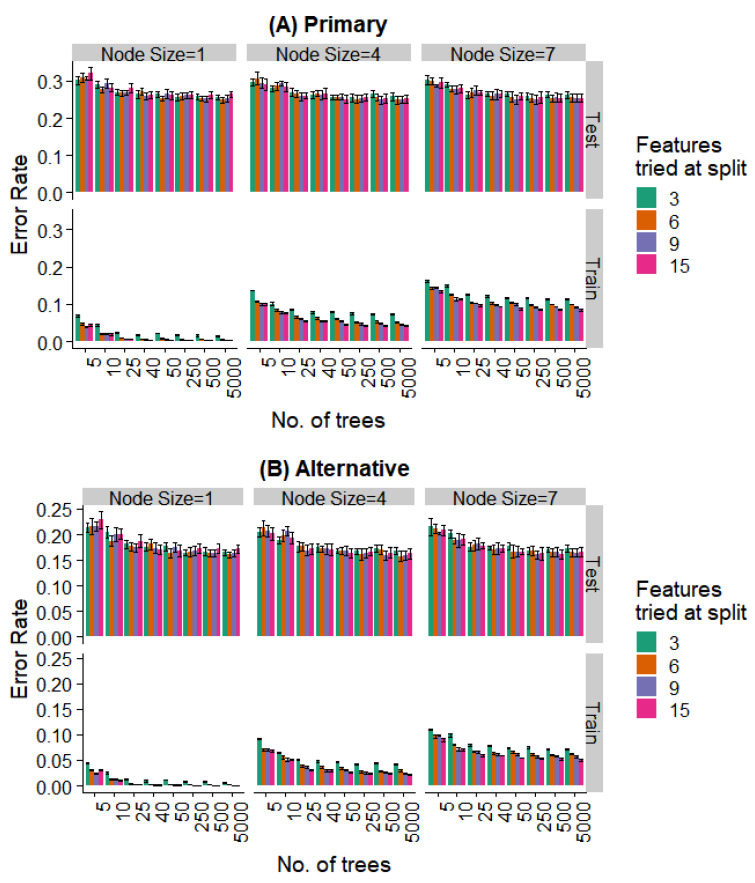
Effect of various training parameters on the random forest model for the prediction of the specific extraction. The minimum node size, features tried at every level of split, and the number of trees are varied and the error rates for the training and test split are plotted. In (**A**), a prediction is considered as an error if it does not agree with the expert’s primary diagnosis, and in (**B**), it is considered an error if the prediction does not agree with the primary or alternative diagnosis.

**Figure 9:**
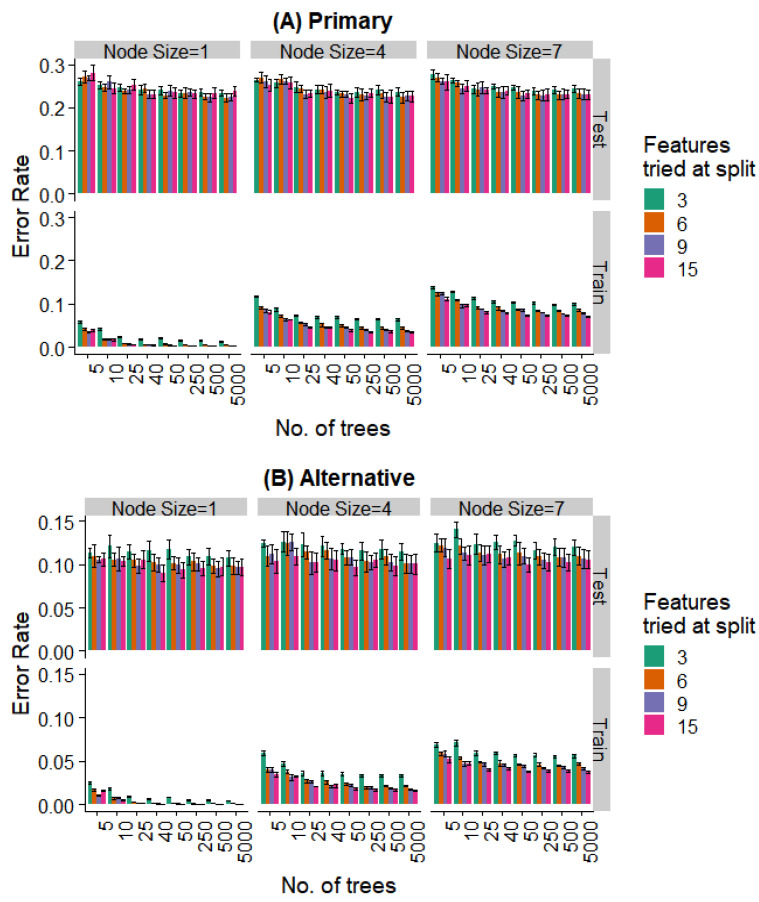
Effect of various training parameters on the random forest model for the binary prediction problem. The minimum node size, features tried at every level of split, and the number of trees are varied and the error rates for the training and test split are plotted. In (**A**), a prediction is considered as an error if it does not agree with the expert’s primary diagnosis, and in (**B**), it is considered an error if the prediction does not agree with the primary or alternative diagnosis.

We varied these hyperparameters during the training of the random forest model. Figure 8 and Figure 9 show the performance against a number of hyperparameters needed to fit the size of the available data. Observing the training data alone, it was evident that (a) performance was better for smaller minimal node sizes as it led to deeper decision trees, (b) the number of features at each split had an initial effect, but this is saturated with increasing feature numbers, and (c) even 50 trees showed a performance statistically indistinguishable from random forests with a much larger number of decision trees. Most notably, the prediction error showed no overfitting in the test data (i.e., no increase in error rate was observed as the complexity of the model increased).

Further, even the relatively weaker hyperparameters (~25 trees, a minimum node size of 4, and 6 features tried at every split) are strong enough to saturate the test set performance, while the training set performance continues to decrease with more complex models. Similar behavior is seen when looking at the prediction of the specific extraction (Figure 8) and the binary problem of predicting extraction vs. non-extraction (Figure 9).

**Figure 10:**
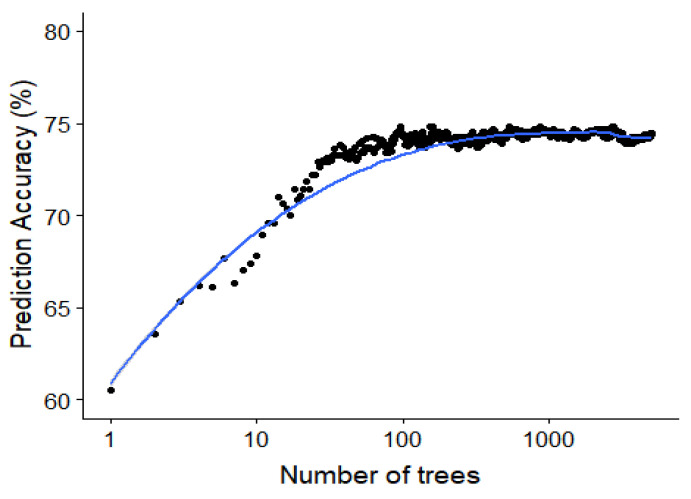
Saturating effect of increasing the number of classifiers in the random forest. The out-of-bag accuracy (an estimate of the test accuracy) plotted against the number of trees for the random forest model predicting the specific type of interaction. This is for a minimal node size of 1 and trying all possible features at every split.

Since the random forest algorithm has an out of bag data sample for the construction of each decision tree, this out of bag error rate can be used to study the effect of adding each additional tree. The out of bag accuracy, which is a proxy for the accuracy on the test set, is visualized in Figure 10 showing the saturation of the performance around 50 to 100 trees in the random forest model.

**Figure 11:**
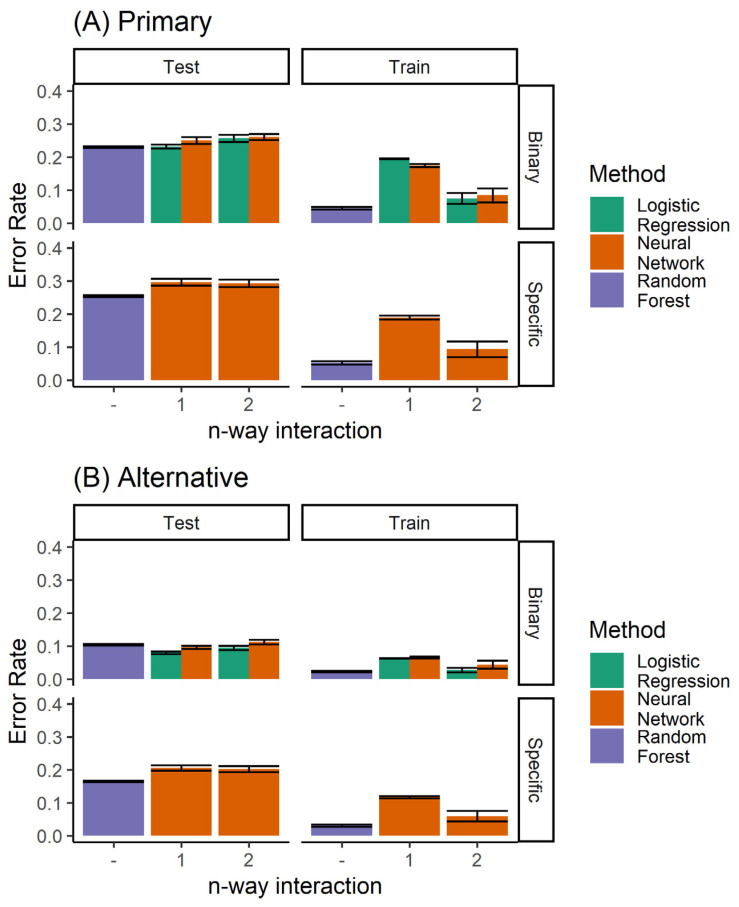
Performance of all the classifiers for predicting (**A**) the primary diagnosis, and (**B**) where agreement with either the primary or the alternative diagnoses is considered to be accurate. Here, both the single and ensemble (random forest) classifiers are included.

When comparing all classifiers (Figure 11), it is clear that the random forest classifier outperforms the neural network model for the prediction of the specific extraction treatment. Logistic regression is able to achieve marginally better performance only for the case of binary prediction when considering both the primary and alternative diagnoses from the expert (top left panel of Figure 8B).

#### 3.2.3. Effects of Individual Features

**Figure 12:**
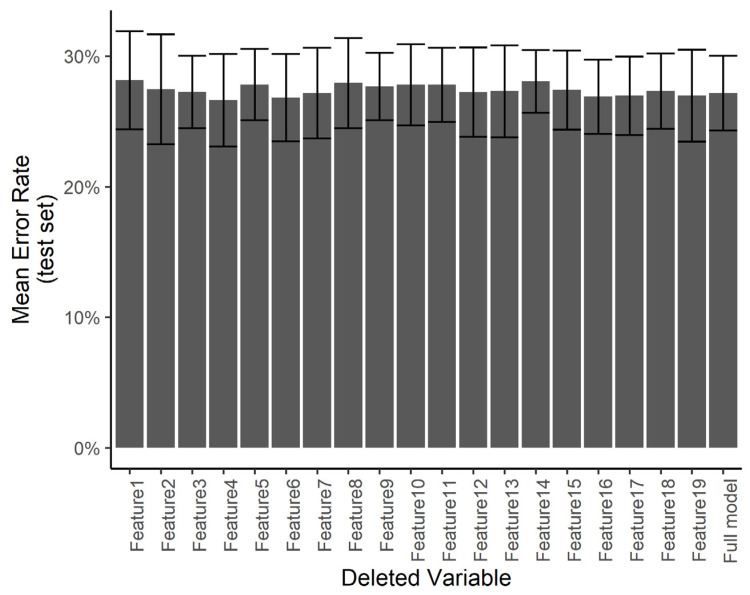
Effect of individual features. The test error for the predicting of the specific treatment plan using the neural network after independently deleting single features from the dataset.

We wanted to understand if certain predictors are especially influential in the treatment decision compared with others. Using a smaller number of predictors would help in minimizing the work required in the clinical setting in order to use the system. However, with a smaller number of predictors, any errors in the measurement of specific predictors may result in a larger error in the final diagnosis. Towards this end, we ran a predictor ablation study on the multinomial regression/neural network model. We measured the effect of deleting each predictor individually on the error rate evaluated over the test set, with a 5-fold cross-validation. Figure 12 shows that no subset of predictors stands out as having a significantly larger effect on the test error. We interpret this to mean that each measurement error in any one predictor is not likely to have a catastrophic effect on the automated diagnosis.

## 4. Discussion

Previous studies have approached this problem by utilizing machine learning using a neural network [9,10]. However, these approaches have been limited due to various shortcomings. The models shown in the results have specifically focused on binary outcomes, i.e., extraction vs. non-extraction, without outlining which tooth or set of teeth needs extraction. Our expert data showed, and it is also generally believed, that this binary decision is a first-order decision, and requires limited expertise when compared with the more resolved decision about which tooth, or a set of teeth, needs to be extracted. Furthermore, the binary decision is determined by fewer parameters (crowding or tooth inclination), a much easier scenario, while a more resolved decision requires the determination of parameters which are yet to be standardized, highlighting the challenges involved in deciding among many other possible outcomes. 

Our research not only focusses on this binary decision but also on the thirteen other possible outcomes which highlight the specific tooth/teeth requiring extraction, creating a new artificial intelligence-based method to predict a plan from among a large number of possible extraction plans (Figure 2) based on the 19 feature elements.

Second, after conducting a thorough review of the existing literature, we limited the diagnostic features to 19 most relevant predictors. The feature vector-elements adopted can be broadly classified into five major categories, i.e., sagittal dentoskeletal, vertical dentoskeletal relationship, transverse dental relationship, soft tissue relationship, and intra-arch conditions. Similar studies [9,10,11] have included many more features which have not only increased their computational requirements but also added redundancy in their data set. Moreover, a small number of features for each patient can be easily obtained from the standard records without utilizing special diagnostic approaches. Fewer features also means that the experts spend less time analyzing the records of each patient, thereby making themselves available to analyze more samples. This helps us evaluate the accuracy of our method in relation to the inter-expert disagreement.

We were able to exploit the diverse features using multiple machine learning models. We limited the number of features for ease of data collection and implementation of the method in the clinic. We were able to extract additional information from the interactions of these features and construct nonlinear predictors for regression using second-degree interactions. Although it resulted in overfitting, regularization was able to generalize the method and counter overfitting. Overall, we observed that ensemble learning and weight regression are both useful in such applications.

One of the limitations of this study was that the treatment outcomes were confined to non-surgical orthodontic procedures only. Further, atypical extraction patterns like lower incisor extraction, second premolar extractions, and extractions dues to pathological reasons, among others, were excluded. In the current optimized model however, the elements that represented such features were not adopted. This is because the current study primarily focused on optimizing routine orthodontic diagnostic protocols.

Finally, though the current model may not yet suffice to achieve complete agreement with human judgments, it should be noted that it has an advantage in that the system can independently improve its prediction accuracy by adding new patient records as templates just as orthodontists might increase their clinical knowledge and experience. This means the model will become more robust clinically for making decisions for individual patient treatment.

We have shown that our limited feature set and machine learning algorithm is able to predict the extraction procedure to an accuracy that is approximately equal to that obtained from different experts. The use of an ensemble classifier (random forest [12]) model allowed us to escape from overfitting, as has been confirmed in many studies earlier [13,14,15]. Further, we have shown that an ensemble of simpler models outperforms more complex models, such as a neural network for our problem. The use of bagged batch training and dropouts may help the neural network model to compete with the random forest model.

A random forest ensemble classifier that simulates orthodontic tooth extraction/non-extraction decision-making was developed and confirmed to show a high performance, within the range of the inter-expert agreement.

The features used for arriving at the extraction or treatment plan in this study can be ascertained by any dentist using this system in the clinic by evaluating the patient. Our future goal is to automatically arrive at these features from X-ray and photographic images. We plan to implement object recognition and landmark detection on images commonly used in the clinical practice. There is some existing work in the field of cephalometric X-ray landmark detection [16,17], and an even more extensive body of work in biomedical image detection [18]. One could also exploit internal features [19,20,21] extracted from image recognition models to predict other diagnostic outputs.

## Figures and Tables

**Table 1:** Percentage agreement on the primary outcome of treatment between different experts.

	Expert 1	Expert 2	Expert 3	Expert 4	Expert 5
**Expert 1**	100.0%	71.1%	64.8%	68.3%	69.0%
**Expert 2**	71.1%	100.0%	70.7%	71.8%	78.0%
**Expert 3**	64.8%	70.7%	100.0%	63.8%	69.7%
**Expert 4**	68.3%	71.8%	63.8%	100.0%	70.4%
**Expert 5**	69.0%	78.0%	69.7%	70.4%	100.0%

**Table 2:** Percentage agreement on either the primary or alternative outcome of treatment between different experts.

	Expert 1	Expert 2	Expert 3	Expert 4	Expert 5
**Expert 1**	100.0%	95.5%	94.4%	95.5%	96.5%
**Expert 2**	95.5%	100.0%	95.5%	95.1%	96.5%
**Expert 3**	94.4%	95.5%	100.0%	93.0%	96.2%
**Expert 4**	95.5%	95.1%	93.0%	100.0%	97.9%
**Expert 5**	96.5%	96.5%	96.2%	97.9%	100.0%

## Supplementary Materials

Supplementary File 1
